# Progress in Surgery

**Published:** 1898-04-16

**Authors:** 


### Progress in Surgery.

### GENITO-URINARY SURGERY.

(*Continued from page* 31.)

**Nephrectomy.**—Mr. J. S. M'Ardle describes what he calls a new operation for nephrectomy. It consists of exposing the kidney by a large T-shaped incision, which is very freely made, but which does not seem to give rise to any subsequent hernia. Transperitoneal examination of the opposite kidney is first made through the horizontal limb of the T, after which the peritoneum is stitched to the median flap and thus protected from infection.[5]

**Nephropexy.**—Jonnesco holds that in order to satisfactorily fix the kidney its whole length should be fixed in the lumbar wound. He has done his method in 10 cases. It consists of the usual incision, removal of excess of peri-renal fat, and exposure of the kidney. Then the kidney, all the soft parts on either side of the wound, and the periosteum of the eleventh and twelfth ribs are transfixed with two or three double sutures, the ends of which are fixed on the skin over rolls of gauze. The stitches are removed on the tenth day.[6]

In all operations on the kidney there is danger of subsequent anuria, to combat which Lennander pursues special methods[7] to increase the blood pressure and reduce the density. He gives subcutaneous injections of normal saline solution both before and after operation—about 1,000 grammes in amount. After operation enemata of 500 grammes of 9 per cent. salt solution are administered, and much water allowed to drink. The opposite loin is covered with a large warm poultice, and the patient carefully protected from cold.

**Surgery of the Ureters.**—Attention is directed by Dr. Randolph Winslow to the liability of the ureters to injury when displaced by abdominal tumours, and to the fact that a few years ago (Van Hook's method was published in 1893) such injury was either fatal or ended in nephrectomy for the relief of the condition. The operation, which he performed with success, consisted of drawing one end of the ureter into the other end, which was laterally split to receive it, by means of traction sutures. Then the whole was carefully sewn together. He quotes a successful case also in the practice of a colleague.[8]

The instruments for catheterising the male ureters and their mode of use are figured by Hurry Fenwick. They are based on the Nitze and Casper models, and the author finds it advantageous at times to colour the urine by ordering fuchsin in pill.[9]

**Renal Tuberculosis.**—An investigation into the results of operations for renal tuberculosis has been made by Dr. Bolton Bangs. The conclusions to which he arrives are that the immediate results of operative treatment are brilliant, even when performed practically *in extremis.* As regards remote results, operation seems to give better prospects than purely hygienic treatment. About 34 per cent. of cases lived for from one to eight years after operation. The operative mortality is 20 per cent. In 25 per cent. the prognosis was good in patients alive from one to nine months after operation, and there were over 50 per cent. surviving and promising cases.[10]

**Surgical Urinary Affections in Children.**—This was the subject of the Lettsomian Lectures by Mr. John Morgan. He lays special stress on the necessity of weighing the possibility of congenital absence of one kidney. With regard to renal sarcomata, he quotes statistics showing the very high mortality of operation for this condition. As regards calculus, he gives preference to litholapaxy for small stones and suprapubic lithotomy for large ones.[11]

**Congenital Genito-Urinary Affections.**—A collection of illustrative specimens was exhibited by Dr. L. R. Sutherland, in Glasgow,[12] chiefly from the Glasgow Children's Hospital. Two were abnormal kidneys lying on the brim of the pelvis near the bifurcation of the aorta. In one case there was entire absence of the right kidney, and in another the kidney was malposed and rudimentary. There were several cases of horseshoe and fused kidneys affected by disease, and of hydronephrosis associated with anencephalism, with dilated bladder, and other conditions. The record is illustrated with some valuable diagrams.

**Nephro-ureterectomy.**—Mr. Henry Morris advocates removal of the ureter, in whole or in part, with the kidney if diseased, and gives several illustrative cases.[13] As a primary operation it is required to prevent, and, as a secondary operation, to cure a fistula. One case comprised tuberculosis of the kidney and ureter, another was a secondary ureterectomy, and the third was for tubercular disease. The conditions are figured in the report.

**Extra-peritoneal Rupture of Bladder.**—A successful case of operation for this condition is recorded by Dr. James Mitchell (Baltimore). It occurred in conjunction with fractured pelvis. The space of Retzius was found filled with blood-stained urine, and on opening the peritoneal cavity no intra-peritoneal rent was discovered. The patient recovered. He appends an analysis of cases to his report, which shows that of those operated on within twenty-four hours 45 per cent. recovered, whereas of those operated on later the mortality was very high indeed, most patients dying within the first week.[14]

**Surgery of the Vas Deferens.**—Reginald Harrison points out that vasectomy may fail to relieve prostatic hypertrophy if the condition be one of fibrosis, and he reiterates the advice not to operate on both sides at one sitting. The effect may not only be great upon the prostate itself, but also upon the kidneys as well, owing to the reduction of back pressure. In all cases the patient should be apprised of the fact t[illegible] t all sexual power will be lost.[15]

**Stricture of Urethra.**—Practical suggestions on the treatment of stricture are given by Dr. Ward Cousins.[16] He has given up forcible dilatation and internal urethrotomy, and adheres to methods of dilatation. He employs tapering catheters with bulbous points and solid, flexible beaks. He advocates the sterilisation of catheters by fixing them to the old Listerian steam-spray apparatus. In chronic cases, if a capillary catheter cannot be inserted, external urethrotomy is advocated as the best measure to adopt, and he believes Wheelhouse's operation will "supersede all the old dodges of finding a way into the bladder without a guide."

